# Familial crossed testicular ectopia: Insights from three sibling cases

**DOI:** 10.12669/pjms.42.(ICON26).15707

**Published:** 2026-04

**Authors:** Anum Liaquat, Syeda Rawash Mehdi, Syed Faisal Usman

**Affiliations:** 1 Anum Liaquat, Department of Pediatric Surgery, Recep Tayyip Erdogan Hospital, 3 km Dera Ghazi Khan Road, Near Mehmood Textile Mill, Muzaffargarh, Punjab, Pakistan; 2 Syeda Rawash Mehdi, Department of Pediatric Surgery, Recep Tayyip Erdogan Hospital, 3 km Dera Ghazi Khan Road, Near Mehmood Textile Mill, Muzaffargarh, Punjab, Pakistan; 3 Syed Faisal Usman, Department of Pediatric Surgery, Recep Tayyip Erdogan Hospital, 3 km Dera Ghazi Khan Road, Near Mehmood Textile Mill, Muzaffargarh, Punjab, Pakistan

**Keywords:** Case series, Crossed Testicular Ectopia (CTE), Cryptorchidism, Inguinal hernia, Diagnostic laproscopy, Genetic factors, Müllerian duct remnants, Siblings, Vas abnormaility

## Abstract

Crossed testicular ectopia is a rare form of urogenital anomalies in which both testes are migrated and descend through a single inguinal canal, one or both testes may be ectopic in the abdomen, the inguinal region or descent to the hemi-scrotum with empty contralateral hemi-scrotum. We report a rare case series of crossed testicular ectopia in three siblings, highlighting the importance of early recognition and treatment of this congenital anomaly. Diagnostic laproscopy and surgical exploration confirmed the diagnosis of crossed testicular ectopia . The patients underwent successful surgical correction, and follow-up showed no complications. This case series suggests a possible genetic component to the etiology of crossed testicular ectopia and emphasizes the need for clinicians to consider this rare condition in patients with abnormal testicular descent.

## INTRODUCTION

Crossed testicular ectopia (CTE) is a rare congenital anomaly where one testis is located into the opposing inguinal canal, or even descend into the opposing hemi-scrotum abdominal cavity. The condition is usually identified during surgery for inguinal hernia or undescended testis or laproscopy,^1^ and fewer than 200 cases have been reported in the literature to date. CTE is commonly associated with other urogenital anomalies, including persistent Müllerian duct syndrome, hypospadias, and scrotal abnormalities. While most reported cases are sporadic, familial occurrence is exceedingly rare, suggesting a possible genetic or hereditary component in selected patients.^2^ CTE can be classified into different types based on the location of the testes.

### Classical (Transverse) Crossed Testicular Ectopia:

Both testes are found in the same scrotal sac, on the opposite side of their normal position. Often seen with inguinal hernias, abnormal gubernacular development, and sometimes with other urogenital anomalies (e.g., hypospadias, persistent Müllerian duct syndrome).

### Intrascrotal Crossed Testicular Ectopia:

Both testes lie within the scrotal sac but are abnormally positioned. They do not cross the midline through the inguinal canal, but instead move in a more lateral position. Similar to the classical type, intrascrotal CTE may also be seen with inguinal hernias or congenital adhesions.

### Intrabdominal Crossed Testicular Ectopia:

One or both testes fail to descend completely and are located in the abdomen on the contralateral side. This is extremely rare and can be seen in severe forms of the condition and can be associated with other conditions such as cryptorchidism, Müllerian duct anomalies^3^-^5^ and urethral anomalies. Associated anomalies of vas deferens are very rare which include fusion defects, supernumerary vas, discontinuity of vas and agenesis of vas.^6^-^8^ The exact embryological mechanism underlying CTE remains unclear. Several theories^9^ have been proposed, including abnormal gubernacular development, fusion or adherence of the developing Wolffian ducts, and mechanical obstruction during testicular descent. Clinical presentation varies depending on age and associated anomalies, but the most common findings include unilateral non-palpable testis with a contralateral inguinal hernia. Preoperative diagnosis is often challenging, and CTE is frequently recognized intraoperatively. Early surgical correction^10^ is recommended to reduce the risks of infertility, testicular malignancy, torsion, and trauma.

**Fig.1:**
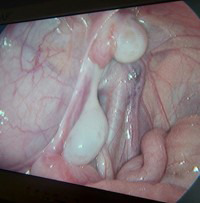
showing cross testicular ectopia on right side with mullerian duct structures (left fallopian tube and rudimentary uterus).

**Fig.2:**
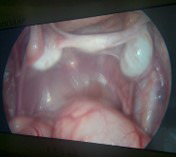
showing cross testicular ectopia on right side with mullerian duct structures (left fallopian tube and rudimentary uterus).

## CASE PRESENTATION

We present a rare case series of crossed testicular ectopia Type-2 in two year-old twin brothers and their one year-old younger sibling with Type-1 along with vas abnormality. On history siblings were born full-term via vaginal birth. No complications occurred during delivery or after birth. Parental consanguinity present. No family history of cryptorchidism, syndromes or exposure to toxins.

**Fig.3:**
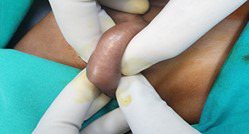
Showing both testes palpable in left hemiscrotum.

The two years old twin brothers presented with a history of bilateral undescended testes. Physical examination revealed underdeveloped empty scrotum and non-palpable testes in the line of descent or any ectopic sites. On ultrasound, only right testes was visualized above deep ring. Diagnostic laparoscopy was performed which showed, right testes above the right deep ring and left testes also on the right side above the right testes. Two separate vas were identified associated with persistent mullerian duct structures including rudimentary uterus and left fallopian tube.

Due to heterogeneous structures (presence of testes, fallopian tube, rudimentary uterus), findings observed and karyotyping was planned, which was XY and bilateral orchidopexy and total excision of mullerian duct structures was done. Postoperative recovery was uneventful. Follow up done at one week, three months, six months, one year showed no long term complications. Their one year old brother presented with right undescended testes and left sided inguinal hernia. On examination, both testes were palpable in left hemiscrotum and left herniotomy and orchidopexy was planned.

**Fig.4:**
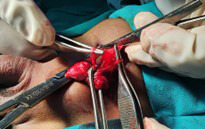
Showing two separate vas and hernial sac excision.

**Fig.5:**
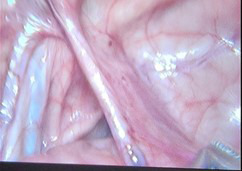
Showing finding of absent vas on diagnostic laparoscopy.

Skin crease incision was made in the left inguinal region. Layers separated, hernia sac carefully identified along with both testes. Hernial sac is dissected free from the surrounding structures, including both vas and any adjacent blood vessels. The sac is excised and ligated.

During herniotomy and bilateral orchidopexy, continuity of both vas deferens near preperitoneal fat was doubtful and could not be appreciated further, for which diagnostic laparoscopy was planned later to see for vas abnormality in addition for diagnosis of mullerian duct remnants. On diagnostic laparoscopy, testicular arteries were identified but both vas and mullerian duct remanats were not found.

Postoperative recovery was uneventful. Follow-up assessments at one week, three months, six months, and one year showed no long-term complications.

## CONCLUSION

CTE is an extremely uncommon anomaly with no confirmed cause. This case series highlights the rare presentation of crossed testicular ectopia . Multiple related anomalies are evident as mullerian duct remnant, vas anomalies, scrotal problems and hypospadias. The familial occurrence of CTE raises important questions regarding potential genetic predispositions or inherited abnormalities in testicular descent, suggesting the need for further genetic evaluation and counseling to understand inheritance patterns. Laparoscopy is useful for both evaluation and management of CTE and associated anomalies. Treatment involves surgically relocating CTE to the orthotopic position. When mullerian duct structures are found, total excision or leaving behind a cuff of mullerian remnants still attached to the testis or the main bulk of mullerian duct structures may be left in situ to preserve testicular blood supply. This case series contributes valuable insights into the management of crossed testicular ectopia and inguinal hernia in a pediatric population, emphasizing the need for genetic investigation, timely surgical intervention, and comprehensive care. The long-term follow-up is essential, particularly in cases of CTE, to ensure continued monitoring of testicular function and overall health.

### Author Contribution:

**AL:** Data collection, Manuscript writing and responsible for accuracy and integrity of the paper.

**SRM:** Literature search and statistical data analysis.

**SFU:** Review and final approval of the manuscript.
